# Case Report: Opposite Effects of *BRAF* Inhibition on Closely Related Clonal Myeloid Disorders

**DOI:** 10.3389/fonc.2021.779523

**Published:** 2021-12-24

**Authors:** Katrin E. Hostettler, Elisa Casañas Quintana, Michael Tamm, Spasenija Savic Prince, Gregor Sommer, Wei-Chih Chen, Thierry Michael Nordmann, Pontus Lundberg, Gregor Thomas Stehle, Thomas Daikeler

**Affiliations:** ^1^ Clinics of Respiratory Medicine, University Hospital Basel, Basel, Switzerland; ^2^ Emergency Department, University Hospital Basel, Basel, Switzerland; ^3^ Pathology, Institute of Medical Genetics and Pathology, University Hospital Basel, Basel, Switzerland; ^4^ Clinic of Radiology and Nuclear Medicine, University Hospital of Basel, Basel, Switzerland; ^5^ Department of Chest Medicine, Taipei Veterans General Hospital, Taipei, Taiwan; ^6^ Department of Dermatology, University Children’s Hospital Zurich, Zurich, Switzerland; ^7^ Division of Hematology, University Hospital of Basel, Basel, Switzerland; ^8^ Department of Rheumatology, University Hospital of Basel, Basel, Switzerland

**Keywords:** case report, Langerhans cell histiocytosis, acute myeloid leukemia, hematopoietic stem cell transplantation, BRAF inhibition, AML—acute myeloid leukemia

## Abstract

Langerhans cell histiocytosis (LCH) commonly co-occurs with additional myeloid malignancies. The introduction of targeted therapies, blocking “driver” mutations (e.g., *BRAF V600E*), enabled long-term remission in patients with LCH. The effect of *BRAF* inhibition on the course and the prognosis of co-existing clonal hematopoiesis is poorly understood. We report on a 61-year-old patient with systemic *BRAF V600E* positive LCH and concomitant *BRAF* wild-type (wt) clonal cytopenia of unknown significance (CCUS) with unfavorable somatic mutations including loss of function (LOF) of *NF1*. While manifestations of LCH improved after blocking *BRAF* by dabrafenib treatment, the *BRAF* wt CCUS progressed to acute myeloid leukemia (AML). The patient eventually underwent successful allogeneic hematopoietic stem cell transplantation (HSCT). We performed an in-depth analyzes of the clonal relationship of CCUS and the tissue affected by LCH by using next-generation sequencing (NGS). The findings suggest activation of the mitogen-activated protein (MAP) kinase pathway in the CCUS clone due to the presence of the *RAS* deregulating *NF1* mutations and wt *BRAF*, which is reportedly associated with paradoxical activation of *CRAF* and hence *MEK*. Patients with LCH should be carefully screened for potential additional clonal hematological diseases. NGS can help predict outcome of the latter in case of *BRAF* inhibition. Blocking the MAP kinase pathway further downstream (e.g., by using MEK inhibitors) or allogeneic HSCT may be options for patients at risk.

## Introduction

Langerhans cell histiocytosis (LCH) is a rare clonal myeloid neoplastic disease characterized by the typical Langerhans-type cells that express CD1a, S100, and CD207 (1). LCH can occur as multisystemic (2) or localized disease (3). About 50% of LCH harbor the *BRAF V600E* mutation, leading to constitutive ligand-independent activation of the mitogen-activated protein kinase (MAPK) signaling pathway, which promotes cell proliferation and survival (4). LCH is associated with other hematological malignancies in 10% of all cases (5), which may present synchronously or later during the course of disease; a common clonal origin has been suggested (4).

The identification of somatic *BRAF V600E* mutation in patients with Erdheim–Chester disease (ECD) provided the rationale for *BRAF* inhibition in the treatment of histiocytosis with altered MAPK pathway (6). In a single-center study, 85% of patients with LCH showed an activating mutation within the MAPK pathway (7). Most often, the activating *BRAF V600E* mutation and more rarely mutations in the tyrosine kinases *NRAS* and *KRAS* have been reported (1, 4). Allogeneic hematopoietic stem cell transplantation is potentially curative in myeloid neoplastic disorders such as LCH. Yet, only few patients and mostly children have received HSCT so far (8, 9).

## Case Description

A 61-year-old European male patient presented with persistent cough, thoracic pain, intermittent dyspnea, and progressive weakness of 3 months’ duration. He was an active and heavy smoker (100 pack-years). Relevant previous medical records included anti-dsDNA-antibody-positive systemic lupus erythematosus (SLE) with skin and joint involvement, mild cytopenia, and complement consumption, well controlled under hydroxychloroquine.

At presentation, neutropenia (0.897 × 10S9/l), monocytosis (0.639 × 10S9/l), thrombocytopenia (80 × 10S9/l), and C-reactive protein (CRP) elevation (93.4 mg/L) were noted. Thoracic CT scan demonstrated disseminated micronodules and few cysts, combined with centrilobular pulmonary emphysema. Lung function was impaired.

**Figure 1:**
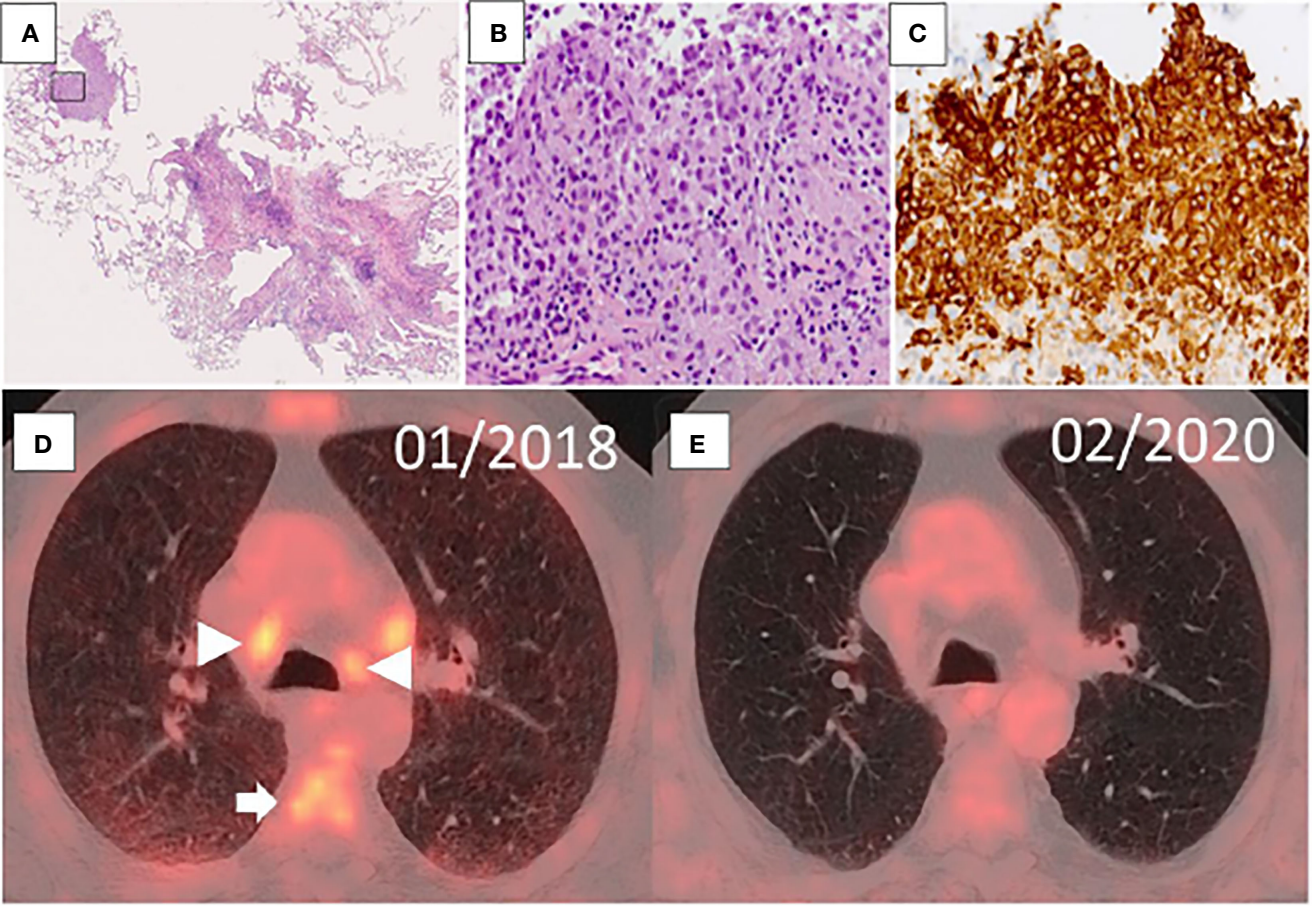
**(A–C)** Transbronchial cryobiopsy of the lung with Langerhans cell histiocytosis. At low power magnification, there are characteristic bronchiolocentric nodules with stellate appearance **(A)**. One nodule starts to form a characteristic cyst (HE; original magnification, 100×). The marked area shows aggregates of Langerhans cells **(B)**, which stain for CD1a by immunohistochemistry **(C)** (**B, C**; original magnification, 200×). **(D, E)** Evolution of imaging findings in FDG-PET/CT over time. FDG-PET at initial diagnosis of LCH (at diagnosis, **D**) shows increased accumulation of FDG in mediastinal lymph nodes (triangular arrow) and bone marrow (arrow), and diffusely distributed across the lungs. The post-therapy scan (13 months later, **E**) shows regression of the lymph nodes in size and normal FDG uptake of lymph nodes, bone marrow, and lung parenchyma.

Histology of transbronchial cryobiopsy was consistent with LCH (**Figures 1A–C**). Sanger sequencing revealed a *BRAF V600E* mutation. Next-generation sequencing (NGS) confirmed the *BRAF* mutation and showed additional mutations in *IDH2*, *ASXL1*, *SRSF2*, and *NF1* (**Table 1**).

**Table 1:** NGS findings in the different tissues.

	At initial diagnosis LCH and CCUS		At diagnosis of AML	
	Mutations	VAF	Mutations	VAF
**Molecular findings LCH**	BRAF V600E	9%	Not applicable	Not applicable
**Lung biopsy**	ASXL1 E850X	10%		
	IDH2 Y179D	23%		
	SRSF2 P95_R102del	No VAF because of low coverage		
	NF1 R1276Q	9%		
**Molecular findings**	BRAF wild type		Not done	
**Peripheral blood**	ASXL1 E850X	20%		
	IDH2 Y179D	23%		
	SRSF2 P95_R102del	33%		
	NF1 R1276Q	22%		
**Molecular findings**	Not done		BRAF wild type	
**Bone marrow**			ASXL1 E850X	47%
			IDH2 Y179D	50%
			SRSF2 P95_R102del	50%
			NF1 R1276Q	49%
			NF1 M546V	48%
			NPM1 W288fs	27%

LCH, Langerhans cell histiocytosis; CCUS, clonal cytopenia of unknown significance; AML, acute myeloid leukemia; VAF, variant allele frequency.

Finally, PET/CT scan demonstrated increased accumulation of 18F-fluorodeoxyglucose (^18^F-FDG) in the spleen, the lungs (diffuse), and bone marrow, and in multiple, enlarged cervical, mediastinal, and abdominopelvic lymph nodes (**Figure 1D**). Therefore, an integrative clinical, radiological, and histologic diagnosis of multisystemic LCH was established.

## Diagnostic Assessment, Details on the Therapeutic Intervention, Follow-Up, and Outcomes

In the light of cytopenia, further diagnostics including NGS of peripheral blood cells were performed, revealing mutations in *IDH2*, *ASXL1*, *SRSF2*, and *NF1* highly predictive for myeloid neoplasm (10, 11). *BRAF*, however, was not mutated (**Table 1**). Morphological assessment of the bone marrow (cytology and histology) a few days later did not show dysplastic changes or excess of blasts and conventional karyotyping was normal, so an integrative diagnosis of CCUS was made. At the time of NGS, no bone marrow DNA was available; thus the analysis was performed from peripheral blood. Since several mutations were observed, and the allelic burden of mutations in the bone marrow and peripheral blood of MDS patients is similar (12), NGS was not repeated from bone marrow DNA. The initial therapy included smoking cessation and oral steroids. Three months later, disease progression of LCH was detected, with worsening pulmonary symptoms and deterioration of oxygenation. Due to the known *BRAF V600E* mutation in the transbronchial cryobiopsy and after a detailed explanation in mutual agreement with the patient, a treatment with the *BRAF* inhibitor vemurafenib was established.

Four weeks after, pulmonary function tests revealed a significant improvement of diffusion capacity with corresponding decrease in dyspnea and improvement of general condition. Accordingly, chest CT scan showed reduced micronodular changes. Due to commonly occurring phototoxic side effects, vemurafenib was replaced by dabrafenib 2 months after.

One month later, the patient presented to the emergency department with fever, generalized weakness, and cough. Peripheral blood showed marked neutrophilia (59.23 × 10S9/L), monocytosis (20.8 × 10S9/L), and blasts (12.5%). Thrombocytes were within normal range, and hemoglobin slightly decreased (126 g/L). In the bone marrow, infiltration of monoblasts was up to 75%, consistent with the diagnosis of AML. NGS of bone marrow revealed an additional *NPM1* mutation and a second *NF1* mutation (**Table 1**).

Dabrafenib treatment was immediately stopped due to the evolution of the CCUS to AML. A cytoreductive therapy with hydroxyurea was initiated. One month later, peripheral blood values were normal, yet the bone marrow still showed an infiltration of monoblasts up to 70%. After two cycles of induction therapy with azacytidine, HSCT from the patient’s HLA-identical sister was subsequently performed.

At the last control, 24 months after transplantation, the patient was in complete molecular remission of his AML. PET/CT also demonstrated a near complete morphological and metabolic remission of the LCH (isolated, residual hypermetabolic cervical lymph node) (**Figure 1E**). His forced vital capacity improved by 700 ml, and arterial oxygen pressure was normal. He suffered an overall moderate graft-versus-host disease but was in a good clinical state.

## Discussion

The detection of *IDH2*, *ASXL1*, *SRSF2*, and *NF1* mutations in both CCUS and LCH clones strongly suggests that both clones originate from a common clonal precursor cell (**Table 1**). The LCH clone additionally acquired *BRAF V600E*, while the CCUS clone did not. Regarding the amount of mutations and the high VAF (20%–30%), it is surprising that the diagnosis of a myeloid neoplasm could not be established initially. It was, however, clear that the mutational profile of this CCUS clone was associated with very high risk of progression (10).

In *BRAF* wild-type cells with *CRAF-BRAF* heterodimers, *BRAF* inhibition (e.g., dabrafenib) can cause paradoxical transactivation of the drug-free promoter leading to extracellular signal-regulated kinase (ERK) activation (13–15). This mechanism is thought to explain why *BRAF* inhibitors are associated with the occurrence of, e.g., squamous-cell skin carcinomas (16). In our patient, there was an additional mechanism that gave the CCUS clone a proliferation advantage over the other BRAF wild-type cells in the bone marrow: At the time of progression to AML, there were two different NF1 mutations with VAF of almost 50%, and both mutations are loss of function (**Table 1**). We assume that these two mutations are placed on the different alleles of NF1 leading to a compound heterozygote state. One major role of NF1 is to negatively regulate RAS proteins through GTPase activity. Thus, biallelic loss of function of NF1 leads to a complete loss of *RAS* suppression resulting in increased proliferation.

Both *BRAF* inhibition triggered paradoxical ERK activation, and synergistic *NF1*-induced *RAS* activation promoted the progression of the CCUS to leukemia. One similar case report portrayed the rapid progression of chronic myelomonocytic leukemia associated with a *RAS* mutation under vemurafenib therapy for malignant melanoma. However, in that case, monocytosis was reversible after stopping *BRAF* inhibition, as the clonal selection was largely dependent on additional paradoxical ERK activation (17).

This is to our knowledge the first case of an AML occurring during *BRAF* inhibition. Because of the possibility of potential paradoxical activation of the MAPK pathway in LCH patients with concomitant clonal hematopoiesis without *BRAF VE600* mutation, a thorough evaluation including molecular diagnostics in cases of blood counts abnormalities before *BRAF* inhibition is warranted. In such cases, inhibition of the MAPK pathway further downstream (e.g., MEK inhibitors) may prevent such an unwanted activation (18).

The successful course of both diseases in our patient after HSCT suggests that a complete renewal of the immune system in patients with severe LCH, with or without concomitant hematological neoplasm, by allogeneic stem cell transplantation may also be a valid treatment option for adult patients.

## Data Availability Statement

The raw data supporting the conclusions of this article will be made available by the authors, without undue reservation.

## Ethics Statement

All patients submitted for allogeneic hematopoietic stem cell transplantation complete extensive general consent forms, attached to their medical records, to the use of their data for scientific purposes. Therefore, permission by the local ethics committee was not required. Nevertheless, the patient was informed in detail about this process and his anonymity is guaranteed.

## Author Contributions

KH analyzed data, helped in drafting the manuscript, and did the interpretation of clinical pulmonary disease related data as pulmonologist. ECQ analyzed data and drafted manuscript and figures. MT gave input in final data analyses and helped drafting the manuscript. SSP performed and interpreted histological diagnostic procedures and gave input in manuscript preparation. GS analyzed longitudinal PET and CT scans and contributed to the drafting of the manuscript. W-CC gave input in initial data analyses and helped drafting the manuscript. PL performed and analyzed molecular genetic tests and helped drafting the manuscript. TN gave input in final data analysis and manuscript preparation. GTS analyzed data and co-drafted the manuscript and the figures. TD initiated and supervised the project, analyzed data, and co-drafted the manuscript.

## Conflict of Interest

The authors declare that the research was conducted in the absence of any commercial or financial relationships that could be construed as a potential conflict of interest.

## Publisher’s Note

All claims expressed in this article are solely those of the authors and do not necessarily represent those of their affiliated organizations, or those of the publisher, the editors and the reviewers. Any product that may be evaluated in this article, or claim that may be made by its manufacturer, is not guaranteed or endorsed by the publisher.

## References

[1] Allen CE Merad M McClain KL. Langerhans-Cell Histiocytosis. N Engl J Med (2018) 379(9):856–68. doi: 10.1056/NEJMra1607548 PMC6334777 30157397

[2] Rodriguez-Galindo C Allen CE. Langerhans Cell Histiocytosis. Blood (2020) 135(16):1319–31. doi: 10.1182/blood.2019000934 32106306

[3] Gupta N Vassallo R Wikenheiser-Brokamp KA McCormack FX. Diffuse Cystic Lung Disease. Part I. Am J Respir Crit Care Med (2015) 191(12):1354–66. doi: 10.1164/rccm.201411-2094CI PMC5442966 25906089

[4] Tran G Huynh TN Paller AS. Langerhans Cell Histiocytosis: A Neoplastic Disorder Driven by Ras-ERK Pathway Mutations. J Am Acad Dermatol (2018) 78(3):579–90.e4. doi: 10.1016/j.jaad.2017.09.022 29107340

[5] Ma J Laird JH Chau KW Chelius MR Lok BH. Langerhans Cell Histiocytosis in Adults is Associated With a High Prevalence of Hematologic and Solid Malignancies. Cancer Med (2019) 8(1):58–66. doi: 10.1002/cam4.1844 30597769 PMC6346231

[6] Haroche J Charlotte F Arnaud L von Deimling A Hélias-Rodzewicz Z Hervier B. High Prevalence of BRAF V600E Mutations in Erdheim-Chester Disease But Not in Other non-Langerhans Cell Histiocytoses. Blood (2012) 120(13):2700–3. doi: 10.1182/blood-2012-05-430140 22879539

[7] Chakraborty R Burke TM Hampton OA Zinn DJ Lim KP Abhyankar H. Alternative Genetic Mechanisms of BRAF Activation in Langerhans Cell Histiocytosis. Blood (2016) 128(21):2533–7. doi: 10.1182/blood-2016-08-733790 PMC5123197 27729324

[8] Veys PA Nanduri V Baker KS He W Bandini G Biondi A. Haematopoietic Stem Cell Transplantation for Refractory Langerhans Cell Histiocytosis: Outcome by Intensity of Conditioning. Br J Haematol (2015) 169(5):711–8. doi: 10.1111/bjh.13347 PMC4433436 25817915

[9] Pan Y Xi R Wang C Fang L Bai J Cai Y. Autologous Hematopoietic Stem Cell Transplantation for Efficient Treatment of Multisystem, High-Risk, BRAF V600E-Negative Langerhans Cell Histiocytosis. J Int Med Res (2019) 47(9):4522–9. doi: 10.1177/0300060519864807 PMC6753533 31426694

[10] Malcovati L Gallì A Travaglino E Ambaglio I Rizzo E Molteni E. Clinical Significance of Somatic Mutation in Unexplained Blood Cytopenia. Blood (2017) 129(25):3371–8. doi: 10.1182/blood-2017-01-763425 PMC5542849 28424163

[11] Zheng G Chen P Pallavajjalla A Haley L Gondek L Dezern A. The Diagnostic Utility of Targeted Gene Panel Sequencing in Discriminating Etiologies of Cytopenia. Am J Hematol (2019) 94(10):1141–8. doi: 10.1002/ajh.25592 PMC9162094 31350794

[12] da Silva-Coelho P Kroeze LI Yoshida K Koorenhof-Scheele TN Knops R van de Locht LT. Clonal Evolution in Myelodysplastic Syndromes. Nat Commun (2017) 8:15099. doi: 10.1038/ncomms15099 28429724 PMC5530598

[13] Poulikakos PI Zhang C Bollag G Shokat KM Rosen N. RAF Inhibitors Transactivate RAF Dimers and ERK Signalling in Cells With Wild-Type BRAF. Nature (2010) 464(7287):427–30. doi: 10.1038/nature08902 PMC3178447 20179705

[14] Su F Viros A Milagre C Trunzer K Bollag G Spleiss O. RAS Mutations in Cutaneous Squamous-Cell Carcinomas in Patients Treated With BRAF Inhibitors. N Engl J Med (2012) 366(3):207–15. doi: 10.1056/NEJMoa1105358 PMC3724537 22256804

[15] McCubrey JA Steelman LS Chappell WH Abrams SL Wong EW Chang F. Roles of the Raf/MEK/ERK Pathway in Cell Growth, Malignant Transformation and Drug Resistance. Biochim Biophys Acta (2007) 1773(8):1263–84. doi: 10.1016/j.bbamcr.2006.10.001 PMC2696318 17126425

[16] Robert C Arnault JP Mateus C. RAF Inhibition and Induction of Cutaneous Squamous Cell Carcinoma. Curr Opin Oncol (2011) 23(2):177–82. doi: 10.1097/CCO.0b013e3283436e8c 21192261

[17] Callahan MK Rampal R Harding JJ Klimek VM Chung YR Merghoub T. Progression of RAS-Mutant Leukemia During RAF Inhibitor Treatment. N Engl J Med (2012) 367(24):2316–21. doi: 10.1056/NEJMoa1208958 PMC3627494 23134356

[18] Nordmann TM Juengling FD Recher M Berger CT Kalbermatten D Wicki A. Trametinib After Disease Reactivation Under Dabrafenib in Erdheim-Chester Disease With Both BRAF and KRAS Mutations. Blood (2017) 129(7):879–82. doi: 10.1182/blood-2016-09-740217 27940476

